# Emergence of π-Magnetism in Fused Aza-Triangulenes: Symmetry and Charge Transfer Effects

**DOI:** 10.1021/acs.nanolett.3c02586

**Published:** 2023-10-23

**Authors:** Jan Patrick Calupitan, Alejandro Berdonces-Layunta, Fernando Aguilar-Galindo, Manuel Vilas-Varela, Diego Peña, David Casanova, Martina Corso, Dimas G. de Oteyza, Tao Wang

**Affiliations:** † Centro de Física de Materiales (CFM-MPC), CSIC-UPV/EHU, 20018 San Sebastián, Spain; ‡ Donostia International Physics Center, 20018 San Sebastián, Spain; § Departamento de Química, Universidad Autónoma de Madrid, 28049 Madrid, Spain; ∥ Institute for Advanced Research in Chemical Sciences (IAdChem), Universidad Autónoma de Madrid, 28049 Madrid, Spain; ⊥ Centro Singular de Investigación en Química Biolóxica e Materiais Moleculares (CiQUS) and Departamento de Química Orgánica, Universidade de Santiago de Compostela, 15782 Santiago de Compostela, Spain; # Ikerbasque, Basque Foundation for Science, 48009 Bilbao, Spain; ∇ Nanomaterials and Nanotechnology Research Center (CINN), CSIC-UNIOVI-PA, 33940 El Entrego, Spain

**Keywords:** aza-triangulene, π-magnetism, Kondo resonance, charge transfer, molecular symmetry, on-surface synthesis

## Abstract

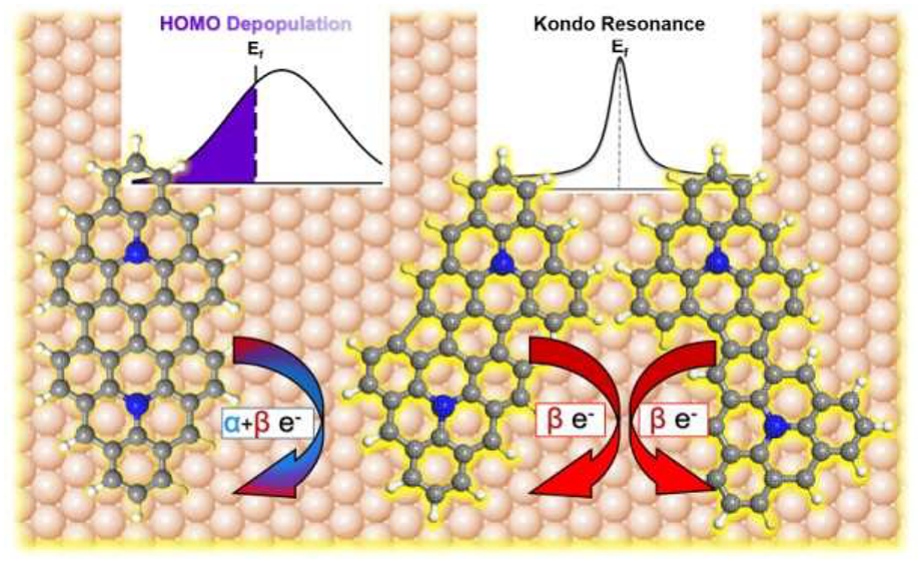

On-surface synthesis has paved the way toward the fabrication and characterization of conjugated carbon-based molecular materials that exhibit π-magnetism such as triangulenes. Aza-triangulene, a nitrogen-substituted derivative, was recently shown to display rich on-surface chemistry, offering an ideal platform to investigate structure–property relations regarding spin-selective charge transfer and magnetic fingerprints. Herein, we study electronic changes upon fusion of single molecules into larger dimeric derivatives. We show that the closed-shell structure of aza-triangulene on Ag(111) leads to closed-shell dimers covalently coupled through sterically accessible carbon atoms. Meanwhile, its open-shell structure on Au(111) leads to coupling via atoms displaying a high spin density, resulting in symmetric or asymmetric products. Interestingly, whereas all dimers on Au(111) exhibit similar charge transfer properties, only asymmetric ones show magnetic fingerprints due to spin-selective charge transfer. These results expose clear relationships among molecular symmetry, charge transfer, and spin states of π-conjugated carbon-based nanostructures.

Mechanistic origins of magnetism in materials can be viewed as breaks in symmetry that allow for unpaired electrons. Though long-time associated with d- and f-shell electrons of metals, unpaired electrons can also emerge in p-shell electrons of carbon-based materials, conferring magnetic properties to π-conjugated structures^1,2^ due to (1) sublattice imbalance^3,4^ in π electrons of the graphene hexagonal lattice that introduce a nonzero net spin, (2) topological frustration^5,6^ of π electrons, preventing double occupation of the same π-bond, and (3) spin polarization^7−11^ of low-energy states driven by Coulomb repulsion or topological phase transitions. Engineering π-magnetism of carbon nanomaterials therefore requires precise control of the most trivial changes in the chemical structure. On-surface synthesis, simultaneously affording atomic precision, inert environment, and substrate stabilization,^12,13^ has proven effective, allowing access to a myriad of π-magnetic molecular materials^1,2^ such as triangulenes,^4,14−16^ graphene nanoribbons,^7,8^ and other graphene nanofragments.^5,9−11,17,18^

Aside from their intrinsic magnetic properties, charge transfer between carbon nanostructures and a metal surface can also generate (or modify) π-magnetism.^19,20^ Adding/removing electron(s) to/from a molecule may modify the spin balance. This requires spin-selective charge transfer: the number of α and β spin electrons transferring to and from the surface must be different. Otherwise, equal contributions would not change the net spin, as in conventional charge transfer scenarios resulting in the depopulation of frontier molecular orbitals.^21−26^ Although each case has been widely reported, a better understanding of parameters influencing the emergence of magnetism vs conventional orbital depopulation by charge transfer could allow their rational tuning.

Triangulene is the most studied molecule displaying π-magnetism due to sublattice imbalance.^27,28^ While solution-phase syntheses of kinetically stabilized derivatives were reported recently,^29,30^ on-surface techniques have produced and characterized larger and smaller derivatives,^14−16,31^ dimers,^6^ trimers,^32^ and larger oligomers.^33,34^ Inspired by how heteroatom doping enriches the chemistry and physical properties of nanographenes, we recently showed that the presence of a nitrogen atom in the carbon framework of triangulene, as in aza-triangulene (**1**, Scheme 1), triggered charge transfer in opposite directions on Au(111) and Ag(111).^35^ Because the redox potentials of **1** lie in the narrow window between the work functions of these substrates,^36^ an oxidized cationic species **1**^**+**^ with open-shell *S* = 1 character was observed on Au(111) while a reduced closed-shell anionic species **1**^**–**^ was observed on Ag(111).^35^ Therefore, **1**, in particular upon fusion into larger derivatives, offers an ideal platform to systematically investigate parameters influencing the spin relevance of charge transfer to and from metal surfaces, as associated with magnetic properties.

**Scheme 1:**
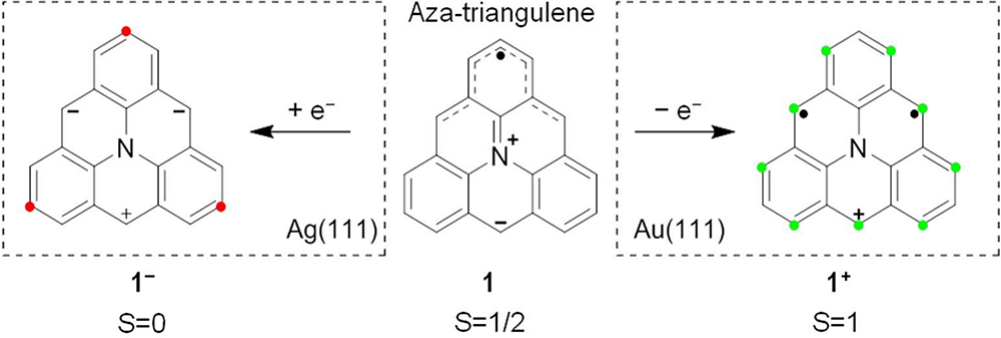
Structure of Aza-Triangulene (**1**) and Its Charge Transfer Properties on Ag(111) and Au(111) On Ag(111), anionic **1**^**–**^ has a closed-shell structure, while on Au(111), **1**^**+**^ has an open-shell structure (*S* = 1). The least sterically hindered sites on the **1**^**–**^ are marked in red. The most reactive carbon sites on **1**^**+**^ as determined by its spin density are indicated in green.

Herein, we fabricate various extended triangulene structures by the fusion of **1** and investigate their tunable magnetic properties. By using high-resolution bond-resolving scanning tunneling microscopy (BR-STM) with a CO-functionalized STM tip,^37,38^ we show that coupling products are random on Ag(111), mostly limited by steric effects, and present closed-shell character. In contrast, on Au(111), we observed fused dimeric products resulting from straight and staggered alignment of reactive carbon sites (those with the highest spin density), producing symmetric and asymmetric structures, respectively. Scanning tunneling spectroscopy (STS), coupled with density functional theory (DFT) calculations, reveals that although all dimers display comparable charge transfer properties, only asymmetric products show magnetic fingerprints. We link their emergence to spin-selective charge transfer^19,20,39^ that results in the formation of singly occupied/unoccupied molecular orbitals (SOMO/SUMO) in the asymmetric products, in contrast with conventional HOMO depopulation^21,25,26^ in the symmetric dimer.

We previously reported the on-surface synthesis and characterization of aza-triangulene (**1**).^35^ Briefly, a ketone-functionalized precursor was deposited on a metal surface, reduced with atomic hydrogen, and annealed to 250 °C. Further annealing at higher temperatures triggered the formation of fused products on both substrates, with lower thresholds observed on Au(111) (∼300 °C) than on Ag(111) (∼350 °C).^35^ Although some molecules maintained one of the ketone groups, in the following, we only focus on aza-triangulene coupling products.

Large-scale STM images on Ag(111) show products without regular fusion patterns or systematically repeating structures (Figure 1a). BR-STM images of planar and thus better identifiable structures (Figure 1a, white and pink arrows) revealed molecules resulting from covalent bonding of aza-triangulene units on their vertices. Such coupling motifs relate to minimal steric hindrance owing to the available space around which other units could approach independently of their relative alignment (Figure 1b and Figure S1). These products did not show any spectroscopic fingerprint in differential conductance (d*I*/d*V*) spectra typically associated with magnetic properties, e.g., zero-bias Kondo resonances, or inelastic spin-flip excitation steps (Figure 1c and Figure S1). This ensemble of results is consistent with the closed-shell nature of **1^**–**^**: (1) high reaction thresholds (350 °C) indicate higher activation energies to fuse stable units of **1**^**–**^, (2) coupling motifs are random, given the even distribution of electronic density of degenerate HOMOs of **1**^**–**^,^35^ only limited by steric considerations, and (3) fusing **1**^**–**^ results in closed-shell dimers.

**Figure 1:**
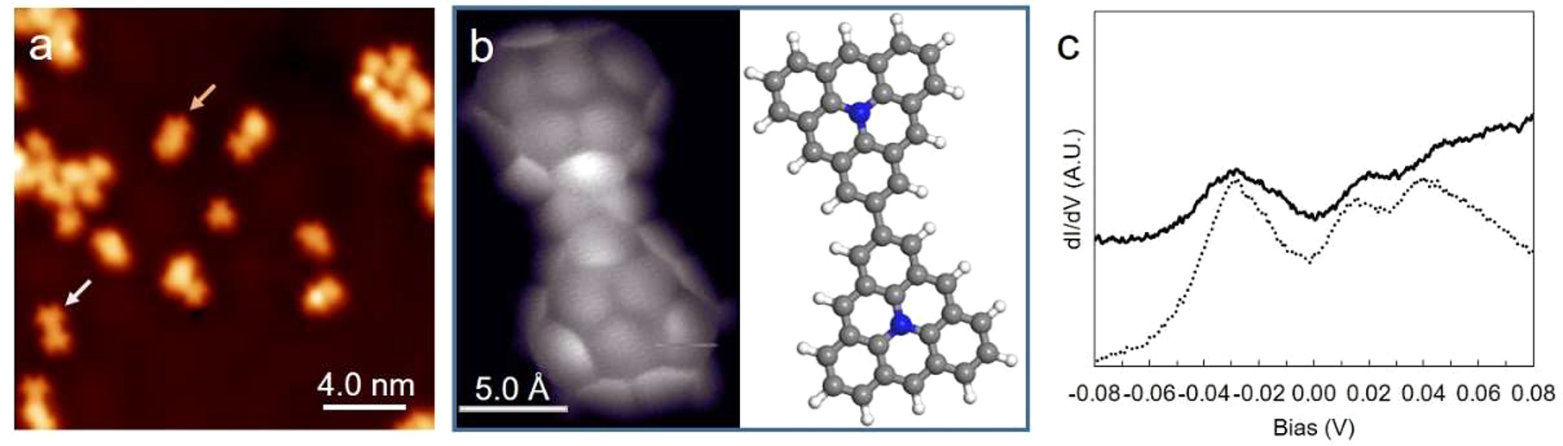
(a) Large-scale STM image of random fusion products on Ag(111) (*U* = −500 mV, *I* = −100 pA). Arrows point to flat dimeric products resulting to fusion of two aza-triangulene molecules (**1**^**–**^). (b) High-resolution bond-resolving STM image of the product pointed out by the white arrow in (a), together with its corresponding structural model. (c) d*I*/d*V* spectra taken on the edge of the dimer shown in (b) (solid line) and on bare Ag(111) (dotted line), showing a lack of signals (other than the Ag(111) surface state) that could be attributed to magnetic fingerprints.

The situation changes on Au(111), where charge transfer in the opposite direction results in **1**^**+**^ with degenerate singly (un)occupied orbitals.^35^ Spin density directly relates to an enhanced reactivity in carbon nanostructures.^1,40,41^ The calculated spin density of **1**^**+**^ (Figure S2) showed that the most reactive sites are carbon atoms on the zigzag edges of this triangular molecule (Scheme 1, atoms in green). This is experimentally confirmed when aza-triangulene units fuse on Au(111). Although large-scale STM images (Figure 2a and Figure S3) show various products, a finite number of systematically repeating structures indicate coupling through preferential carbon sites. BR-STM images revealed the most common dimeric product, **2** (Figure 2a; blue arrow), formed by coupling three reactive carbon atoms (Figure 2b; green circles) of two symmetrically aligned monomeric units. This structure accounting for more than half of the fused products is consistent with the electronic structure of **1**^**+**^, since the activation energy to couple carbons harboring reactive radicals is typically very low.^42^ Additionally, approaching three pairs of neighboring carbon atoms should have been sterically prevented if not for the thermodynamic gain from quenching reactive sites of the monomer, which following the Bell–Evans–Polanyi principle also lowers the kinetic barrier.^43,44^

**Figure 2:**
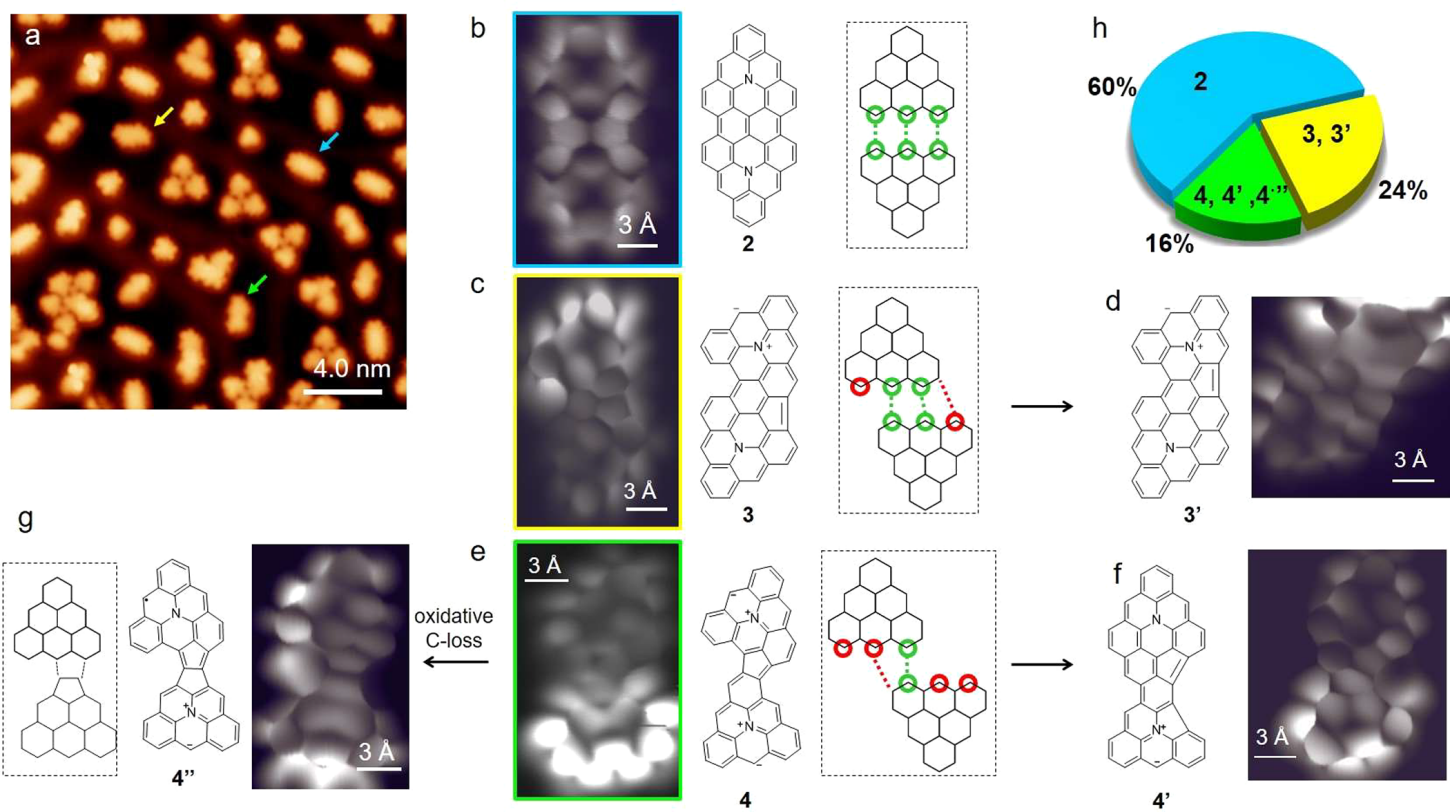
(a) Large-scale STM image of products on Au(111) (*U* = −500 mV, *I* = 100 pA). (b–g) Structure and high-resolution bond-resolving STM images with CO-functionalized tip acquired at constant height. Blue, yellow, and green arrows point to the rectangular, rhombic, and trapezoidal structures, respectively. (b–g) Scheme of bonding alignment, BR-STM images, and chemical structures of fused products. (h) Distribution of products from a statistical analysis of approximately 100 fused products (see Table S1 for details).

Other main products show rhombic (Figure 2a; yellow arrow) and trapezoidal (Figure 2a; green arrow) shapes. BR-STM images of the rhombic species revealed either of two structures **3** and **3′** (Figure 2c,d), the latter of which can be thought of as a rearrangement of the former. Meanwhile, BR-STM images of the trapezoidal species revealed one of the structures **4**, **4′**, and **4″** (Figure 2e–g). **4′** can be thought of as a rearrangement product of **4**, while **4″** results from the oxidative loss of a carbon atom from **4**. We note that rearrangement reactions^45−47^ and oxidative carbon loss^48^ have been previously observed in the on-surface synthesis of other carbon-based nanostructures. For practical reasons, we used large-scale STM images to report yields of formation collectively. Only BR-STM imaging with a CO-functionalized tip could distinguish **3** from **3′** (they differ only in the position of the five-membered ring) or **4** from **4′** and **4″**. Additionally, BR-STM could reveal hydrogenated derivatives, indistinguishable from fully π-conjugated products without CO functionalization (the former requiring tip-induced deprotonation to transform into the latter, as illustrated for **4** in Figure S3). For these reasons, we grouped the products into rhombic (**3**, **3′**) and trapezoidal (**4**, **4′**, and **4″**) species in the statistics, constituting almost a fourth and a sixth of the fused products, respectively (Figure 2h).

We interpret such a distribution by considering how monomeric units fused into dimers. **3** and **4** result from a staggered alignment of two monomeric units (Figure 2c,e), departing from their symmetric alignment in **2** (Figure 2b). Forming one more covalent bond to produce **3** makes it a more exothermic product than **4**, justifying the higher yield of the rhombic structures compared to trapezoidal structures. For **3**, two of the bonds are through pairs of reactive sites of the monomer (Figure 2c, green lines), while the other (red line) is between a reactive carbon and the vertex of another. Meanwhile, for **4**, two bonds are formed: one is through a pair of reactive carbon atoms (Figure 2d, green line), and another (red line) is through a reactive carbon and the vertex of another molecule. Thus, the yields of fused products (**2** > **3** > **4**) are dictated by the number of effectively “quenched” high-energy reactive carbon atoms bonded to those of another monomer. It follows that some reactive sites of a monomeric unit are not bonded to a reactive carbon of another unit (Figure 2c,e, red circles), so that resonance structures of **3** and **4** may be written (Figure S5) to harbor π-radicals (i.e., explicitly showing open-shell structures). Nevertheless, this open-shell structure can be avoided by drawing these molecules with a zwitterionic pyridinium moiety, as proposed previously for **1** (see Figure S5).^35^ Moreover, DFT calculations supported a closed-shell ground state for charge neutral **3** and **4**.

Differential conductance (d*I*/d*V*) spectra of the symmetric dimer **2** revealed resonances at −1.25 V, 5 mV, 460 mV, and 860 mV (Figure 3a,b). Figure 3b shows a short-range spectrum around the Fermi level. d*I*/d*V* maps obtained with a CO-functionalized tip at these energies (Figure 3c) show qualitative agreement with DFT-calculated density of states (DOS) of the HOMO-1, HOMO, LUMO, and LUMO+1 (Figure 3d; see Figure S6 for orbital wave functions). Nevertheless, apparent discrepancies between the calculated DOS and experimental d*I*/d*V* maps appear, mainly due to the p_*x*_p_*y*_-wave character of the CO-functionalized tip.^49,50^ A better fit is thus obtained with simulated d*I*/d*V* maps considering tunneling through p-orbitals of the CO-functionalized tip (Figure 3e).^49,50^ The resonance corresponding to the HOMO being centered slightly above the Fermi level (Figure 3b) is indicative of its partial depopulation, i.e., charge transfer from **2** to the Au(111) substrate. This agrees with periodic DFT calculations that predict a charge transfer from **2** to Au(111) of ∼1.4 electrons (see the Supporting Information for computational details and Figure S7 for the calculated density of states distribution). Henceforth we write **2**^**+**^ to emphasize charge transfer to the surface and the cationic molecular nature.

**Figure 3:**
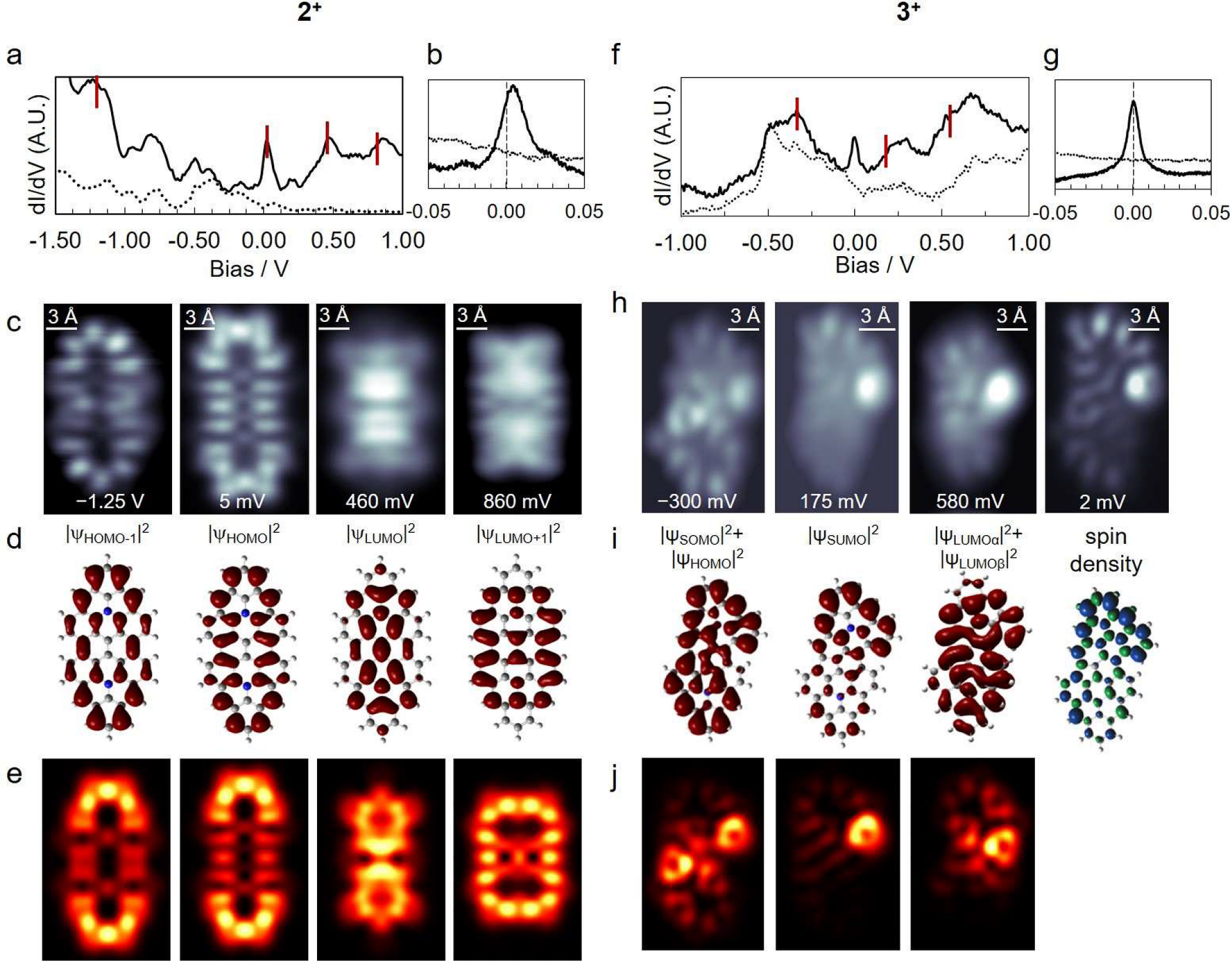
Long-range (a, f) and low-energy (b, g) conductance spectra taken on Au(111) (dotted lines) and on dimers **2**^**+**^ and **3**^**+**^ (solid lines). (c, h) dI/dV maps at the designated energies acquired at constant height mode. (d, i) DFT-calculated DOS of dimers **2** and **3**^**+**^ in the gas phase ((i) includes spin density for **3**^**+**^). (e, j) Simulated dI/dV maps for the orbitals of **2**^**+**^ and **3**^**+**^ listed and shown in (c) and (d). The simulations consider a CO-functionalized probe (see details in the Supporting Information).

Relaxation of the asymmetric structures **3** and **4** on Au(111) similarly predicts a charge transfer of 1.11 and 1.15 electrons, respectively (Table 1 and Figure S8). Indeed, similarly to **1**,^35^ the calculated oxidation potentials of **2**–**4** were all in the same range (5.0–5.2 eV) and lower than the work function of the Au(111) substrate (∼5.3 eV). However, in contrast to the closed-shell product **2**^**+**^, asymmetric products **3**^**+**^ and **4**^**+**^ clearly manifest magnetic fingerprints (in particular a zero-bias Kondo resonance) associated with singly occupied molecular orbitals that result from their cationic nature (Figure 3f–j).

**Table 1:** Contribution of α and β Electrons to the Charge Transfer from the Molecule to Au(111)

molecule	charge transfer α + β	magnetization (α – β)	α contribution	β contribution	% β/% α
**2**	1.4	0.002	0.699	0.701	50/50
**3**	1.11	0.77	0.17	0.94	85/15
**4**	1.15	0.95	0.10	1.05	91/9

Figure 3f shows a representative d*I*/d*V* spectrum acquired on **3** while Figure 3h shows conductance maps of the various resonances, in particular, at the energies marked with red lines. Whereas experimental findings cannot be reproduced with calculations of neutral **3**, a reasonable agreement is readily achieved with DFT-calculated DOS of **3**^+^ in the gas phase (Figure 3i; see Figure S9a for wave functions of associated states). We identify the peak at −300 mV with a convolution of the HOMO and SOMO, the 175 mV peak with the SUMO, and the 580 mV peak with the LUMO. Additionally, the spatial distribution of the Kondo resonance (Figure 3h), which can be fitted by a Frota function^51^ with a full width at half-maximum (fwhm) of 8.6 mV (Figure S10), is consistent with the SOMO and also matches the calculated spin density of **3**^+^ (*S* = 1/2, Figure 3i). Discrepancies between d*I*/d*V* maps and calculated DOS could be due not only to tunneling via the CO tip^49,50^ but also to molecular distortions between the structure in the gas phase and that adsorbed on Au(111) (Figure S9). Simulated d*I*/d*V* maps that match the experimental data (Figure 3j) were generated by considering tunneling between the CO-functionalized tip and the relaxed structure of **3**^+^ adsorbed on Au(111) (Figure S9; see the Supporting Information for computational details).

Similarly to **3**, **4** presents d*I*/d*V* spectra and maps that match the electronic structure of its cation **4**^+^ with an *S* = 1/2 state (Figures S9c,d and S11). We note that tip-induced dehydrogenation (Figure S4) provides further proof of charge transfer.^17,35,52^ These findings on the different dimers are similar to those of **1**, in which removing one electron was necessary to reproduce electronic structures measured on Au(111).^35^ The low barrier to charge transfer for **1** was confirmed recently by studies illustrating the ease of oxidation of derivatives synthesized in solution.^53,54^ Interestingly, monomers and dimeric and even trimeric fusion products (see Figure S12) result in comparable charge transfer regardless of their increasing size. We further contrast our findings with the lack of charge transfer in all-carbon triangulene^4^ and its dimer derivative^17^ as a clear example of how nitrogen doping enriches the chemistry of graphenic nanostructures.^25,26,55−57^

Symmetric and asymmetric dimers manifest different magnetic behaviors on Au(111). Whereas in **2**^**+**^ charge transfer induces the partial depopulation of the HOMO, in **3**^+^ and **4**^+^ electron transfer results in the emergence of SOMO/SUMO and an associated Kondo resonance as typical magnetic fingerprints. Indeed the calculated magnetization was found to be almost 0 for **2**^**+**^ but close to 1 for **3**^+^ and **4**^+^ (0.77 and 0.95, respectively) (Table 1). **2**^**+**^ does not present magnetic fingerprints because of equal contributions of α and β electrons to charge transfer, while **3** and **4** essentially transfer a β electron to the Au(111) surface, turning **3**^+^ and **4**^+^ into open-shell radicals.

Charge-transfer-related magnetism is promoted by (i) transfer of an electron (as close as possible to unity), (ii) a narrow state width, and (iii) large Coulomb repulsion.^1,58^ All these disfavor **2**^**+**^: (i) it departs most from a pure single electron transfer scenario (1.4 electrons), (ii) its planarity promotes better coupling with the substrate, resulting in stronger hybridization and thereby larger state width than the nonplanar **3**^+^ or **4**^+^ (relaxed 3D morphologies of **2**, **3**, **3′**, **4**, and **4′** on Au(111) as calculated by DFT are shown in Figure S13 for comparison), and (iii) Coulomb repulsion is much lower, as evidenced by gas-phase DFT calculations on cationic radical **2**^+^ (Figure S14), resulting in a SOMO–SUMO gap 1 eV lower than that for **3**^+^ (Figure S9a). The lower Coulombic repulsion is due to wider π-conjugation in **2**^**+**^,^1,8,41,59^ tied to its symmetric structure.^18^ The HOMO of **2** is globally delocalized and distributed uniformly on the molecule (Figure S6). We note that it is not symmetry *per se*, but the undisrupted π-conjugation that ultimately prevents magnetization of **2**^**+**^. In contrast, the asymmetric bonding configuration of **3** and **4**, bearing five-membered rings, that disrupts the π-conjugation of the hexagonal lattice in a way that carbon atoms from the same sublattice form a π-bond, leading to unfavorable spin frustration.^60^ Therefore, the HOMO of asymmetric products tends to localize to one subunit. Electrostatic forces scaling inversely with distance, more localized states in asymmetric dimers display stronger Coulomb repulsion^1,61,62^ leading to spin-selective charge transfer. That is, the partially depopulated HOMO splits into SOMO and SUMO (gapped by Coulomb repulsion) to lower the total energy of the system.

While **3** and **4** break the symmetric alignment of **2**, they remain fused dimeric pairs of equivalent monomeric units. Breaking the symmetry further in **4″** by coupling different monomeric units (Figure 2g) allowed generating a high-spin *S* = 1 system. **4″** can be found as the doubly hydrogenated **4″-2H**, with BR-STM unambiguously revealing the hydrogenated rings by their larger size and characteristic contrast (Figure 4a). Despite the odd number of sp^2^ carbons (odd π-electrons) **4″-2H** does not display a Kondo resonance (Figure 4b). However, after tip-induced dehydrogenation to **4″-H** (Figure 4c) that adds one electron to the π-system, it displays a strong and narrow Kondo resonance peak (fwhm = 9.1 mV, Figure S8) typical of *S* = 1/2 systems. As in previously discussed molecules, including **1**, experiment and theory could only be reconciled by considering the molecules in their cationic states. Kondo resonance maps obtained at *U* = 5 mV match well the calculated spin density of the cationic **4″-H**^**+**^ (*S* = 1/2) (Figure 4d). Upon further conversion to **4″** (Figure 4e), i.e., adding another π-electron, the spectrum transforms to a much weaker and wider peak (fwhm = 15.0 mV, Figure S10), typical of *S* = 1 systems.^17,35,61,63^ The Kondo maps are consistent with the calculated spin density of **4″**^**+**^ with the *S* = 1 ground state (Figure 4f). In addition, d*I*/d*V* maps of the molecular resonances match DFT calculations on the cationic *S* = 1 **4″**^**+**^ species, confirming the presence of two pairs of SOMOs/SUMOs (Figure S15). *A priori* this seems surprising, as one would expect that neutral **4″** (with its odd number of electrons and *S* = 1/2) would transform to a zero-spin ground state upon charge transfer to Au(111) in **4″**^**+**^. This scenario results from the unpaired electron in **4″** being in an orbital (SOMO) of lower energy than the HOMO (Figure S16). Thus, upon charge transfer, the substrate abstracts an electron from the HOMO, resulting in **4″**^**+**^ with two unpaired π-electrons (*S* = 1). The completely broken molecular symmetry directs spin-selective charge transfer that leads to a high-spin ferromagnetic system.

**Figure 4:**
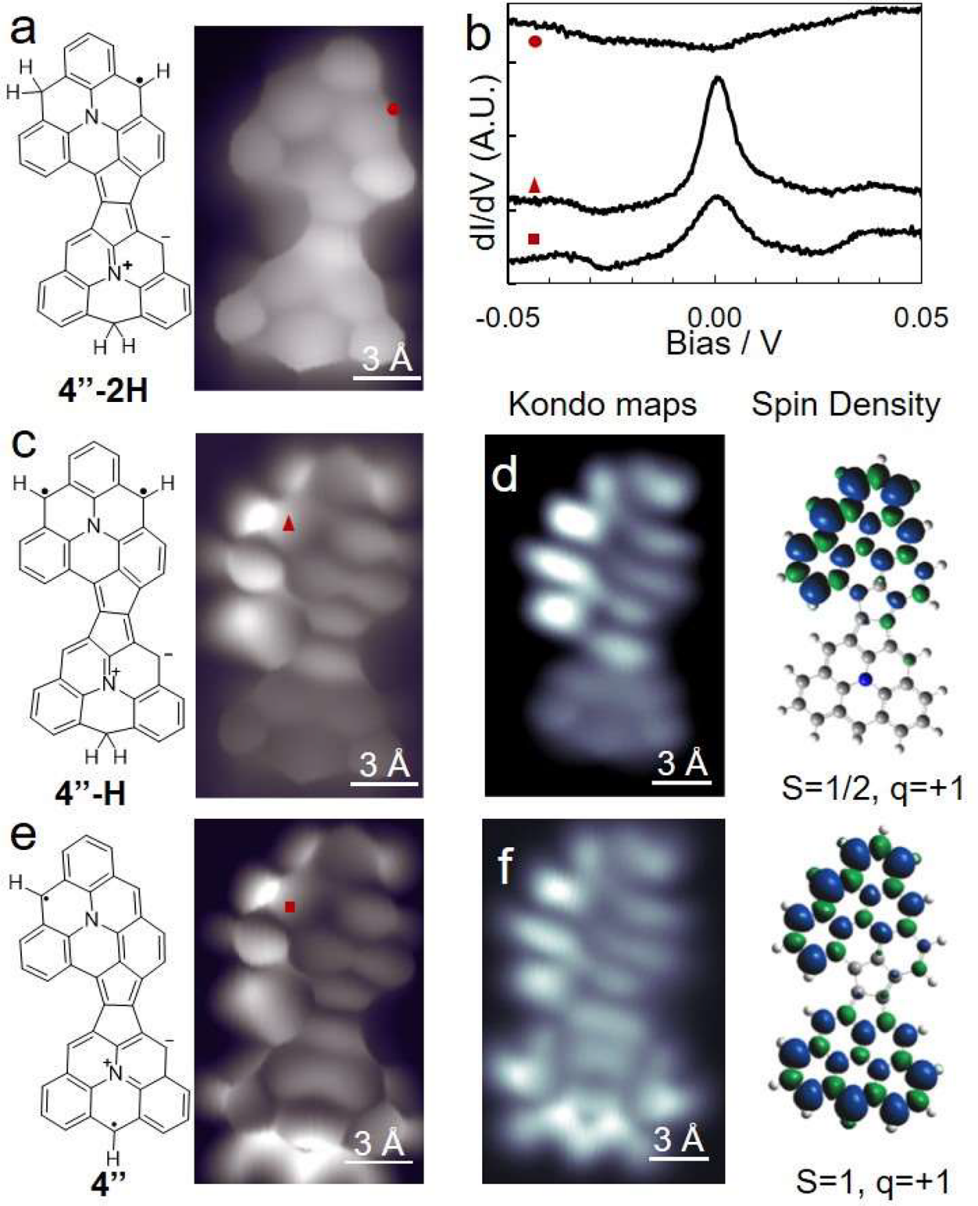
(a) Structure and BR-STM image of doubly hydrogenated dimer **4″** (**4′-2H**). (b) Low-energy spectra of **4″-2H**, **4″-H**, and **4″** on Au(111). (c,e) Structure and BR-STM image of (c) **4″-H** and (e) **4″**^•^. (d,f) Constant height STM images acquired at 5 mV corresponding to Kondo maps and calculated spin density (on the right) of (d) **4″-H**^**+**^ and (f) **4″**^**+**^. (a, c, e) Only hydrogen atoms bonded to sp^3^ carbons or to sp^2^ radical sites are explicitly shown in the resonant structure. All STM images were acquired at constant height.

In summary, we relate charge transfer and symmetry considerations to the chemical and magnetic properties of aza-triangulene and its fused derivatives. We show how the molecule’s spin density (or its absence) determines its reactivity. In the closed-shell **1**^**–**^ (on Ag(111)), the dimerization processes require a higher activation temperature and result in random products, mainly limited by steric effects. In contrast, **1**^**+**^ with the *S* = 1 ground state (on Au(111)), displays an enhanced reactivity, with a lower thermal activation threshold and driven by spin density, consequently with a more limited number of products. All the fused products on Au(111) reveal comparable molecule-to-substrate electron transfer but with disparate effects on the molecule’s magnetism: whereas the symmetric **2**^**+**^ displays a closed-shell structure, the asymmetric **3**^**+**^ and **4**^**+**^ unambiguously display magnetic fingerprints. **2**^**+**^ is fully planar (promoting electronic coupling with the metallic substrate, leading to wider orbitals) and has a well-extended π-conjugation (promoting a reduced Coulomb repulsion), two factors that disfavor magnetism. In contrast, the nonplanarity of the asymmetric products reduces their hybridization with the substrate. Besides, asymmetric bonding configurations disrupt π-conjugation, causing electrons to localize and thus suffer stronger Coulomb repulsion. In turn, adopting single-electron occupancies on particular orbitals becomes favorable, leading to magnetic fingerprints. Lastly, further symmetry breaking by coupling two disparate monomers could lead to high-spin systems. This study shows how charge transfer and symmetry considerations could synergistically affect magnetic properties toward the future design of π-magnetic nanostructures.

## Supporting Information Available

STM/STS data of additional dimers on Ag(111), spin density and Kondo map of **1**^**+**^, large-scale images of fused products on Au(111), tip-induced deprotonation experiments of **4**, resonance structures of **3** and **4**, molecular orbitals of **2**, density of states for **2**, **3**, and **4** adsorbed on Au(111), molecular orbitals of **3**^**+**^ and **4**^**+**^, comparative Kondo resonances of the various probed molecules, STS point spectra and experimental and simulated conductance maps of **4**^**+**^, STM and STS analysis of trimers, relaxed structures of molecules adsorbed on Au(111), calculated molecular orbitals of **2**^**+**^, STS point spectra, conductance maps, and molecular orbitals of **4″**^**+**^, frontier molecular orbitals of **4″** before and after charge transfer, methods description of STM measurements, and DFT calculations (PDF)
