# Supplementary material for: Emergence of π-Magnetism in Fused Aza-Triangulenes: Symmetry and Charge Transfer Effects
Source: Nano Lett. 2023 Oct 23;23(21):9832–40. doi: 10.1021/acs.nanolett.3c02586 (PMC10722538; doi:10.1021/acs.nanolett.3c02586)
Supplement: Supplementary file 1 — nl3c02586_si_001.pdf [file nl3c02586_si_001.pdf]

Emergence of π-Magnetism in Fused Aza-Triangulenes: Symmetry and Charge Transfer Effects

Jan Patrick Calupitan,[1,2,,‡,†] Alejandro Berdonces-Layunta,[1,2,†] Fernando Aguilar-Galindo,[3] Manuel Vilas-Varela,[4] Diego Peña,[4] David Casanova,[2,5] Martina Corso,[1,2] Dimas G. de Oteyza,[1,2,6,*] Tao Wang,[1,2,*]*

[1] Centro de Física de Materiales (CFM-MPC), CSIC-UPV/EHU, 20018 San Sebastián, Spain

[2] Donostia International Physics Center, 20018 San Sebastián, Spain

[3] Departamento de Química, Universidad Autónoma de Madrid, 28049 Madrid, Spain

[4] Centro Singular de Investigación en Química Biolóxica e Materiais Moleculares (CiQUS) and Departamento de Química Orgánica, Universidade de Santiago de Compostela, 15782 Santiago de Compostela, Spain

[5] Ikerbasque, Basque Foundation for Science, 48009 Bilbao, Spain

[6] Nanomaterials and Nanotechnology Research Center (CINN), CSIC-UNIOVI-PA, 33940 El Entrego, Spain

[‡] Current address : Sorbonne Université, CNRS, Institut Parisien de Chimie Moléculaire, IPCM, F-75005 Paris, France

[†] These authors contributed equally

*jan.calupitan@sorbonne-universite.fr; *d.g.oteyza@cinn.es; *taowang@dipc.org

Supplementary Figures

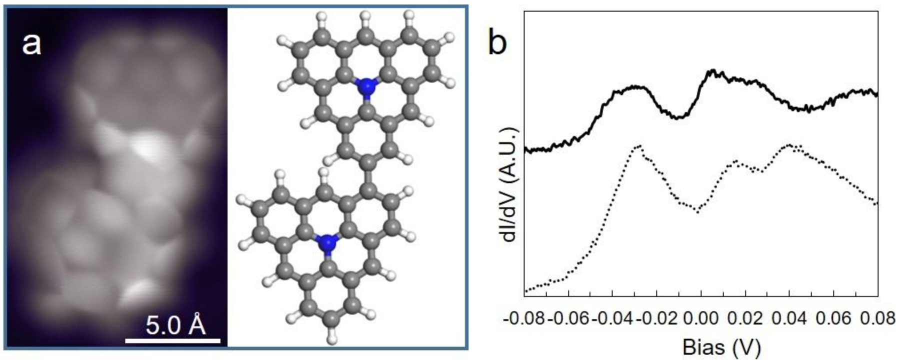

Figure S1. High-resolution BR-STM of the product pointed at by the yellow arrow in Figure 1a (main text), along with its structural model. dI/dV spectra taken on this molecule (solid line) *vs.* on Ag(111) (dotted line).

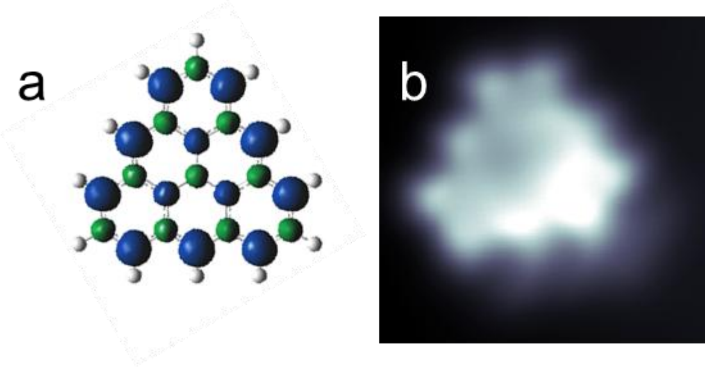

Figure S2. (a) DFT calculated spin density distribution of positively charged aza-triangulene. (b) Experimental Kondo current map of aza-triangulene taken at a bias of 2 mV on Au(111).

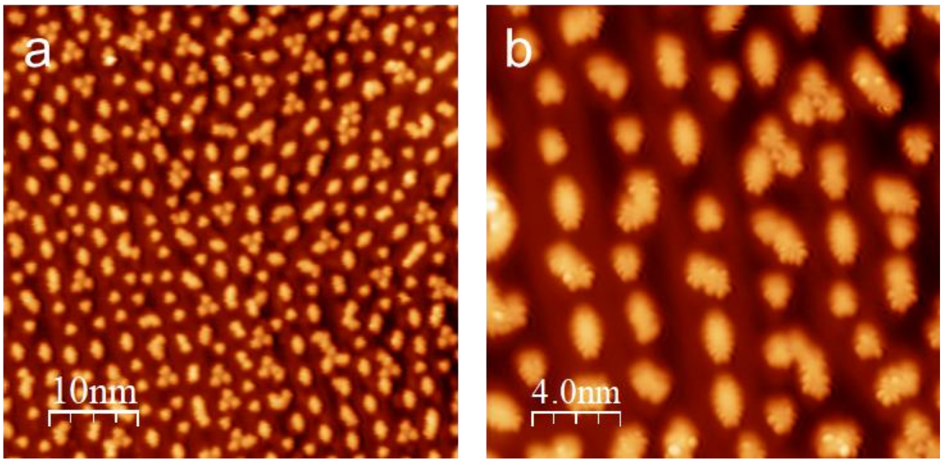

Figure S3. Large-scale STM images of fused products on Au(111).

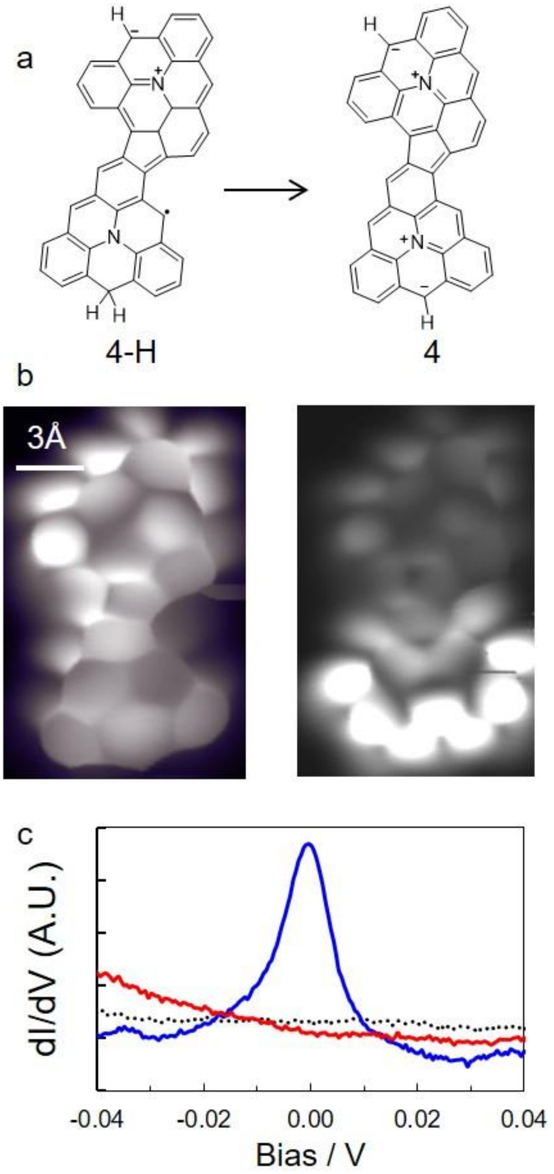

Figure S4. (a) Tip induced deprotonation reaction[1] from **4-H** to **4**. (b) BR-STM images before and after deprotonation. (c) dI/dV spectra obtained before (red) and after (blue) deprotonation. Dotted line is the reference on bare Au(111) surface.

4-H has an odd number of π-electrons in its neutral form but did not display any magnetic fingerprint anywhere on the molecule. A strong Kondo resonance (associated with S=1/2) appears after removing the extra hydrogen by tip-induced manipulation to form **4**, which in its neutral form has been predicted to be of closed-shell character. In both cases, experiment and theory can be reconciled by removing an electron from the molecules. That is, we are probing the cationic species **4-H**$^+$ with a closed-shell structure and **4**$^+$ with a S=1/2 state.

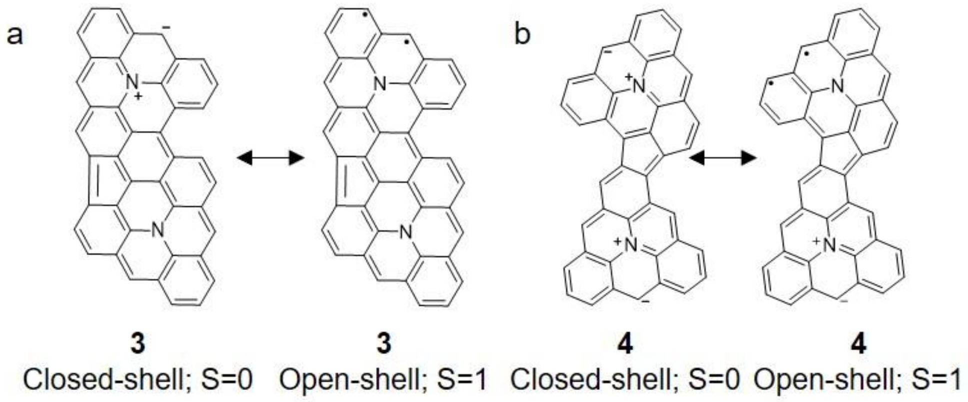

Figure S5. Resonance structures between zwitterionic and diradical forms of (a) **3** and (b) **4**. DFT optimizations (Gaussian 16, M062X/6-311g(d,p)) show that neutral **3** and **4** have closed-shell ground states. The triplet state S=1, which could be assigned with resonance structures that harbor the diradicals, were found to be 0.32 eV and 0.19 eV higher in energy for **3** and **4** respectively. The closed-shell character of the S=0 state was confirmed by performing a stability check of the wavefunction after optimization by using the *stable=opt* keyword.

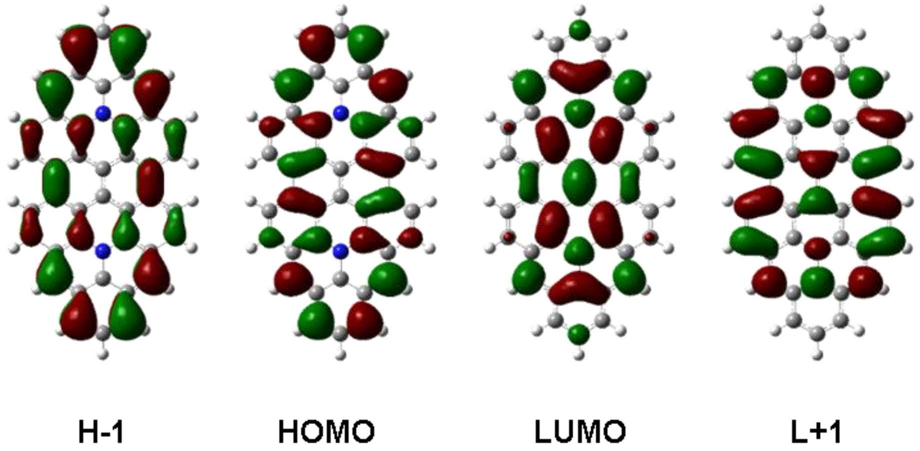

Figure S6. Molecular orbitals generated from the optimized structure of **2** by DFT. Using the functionals M062x or B3LYP and basis set 6-311g(d,p) produced the same shapes of orbitals. The respective energies from L+1 to H-1 were 2.72, 2.55, 0, and -1.02 eV for the M062x functional while 0.855, 0.455, 0, and -1.26 eV for the B3LYP functional (with the HOMO set to 0 eV.

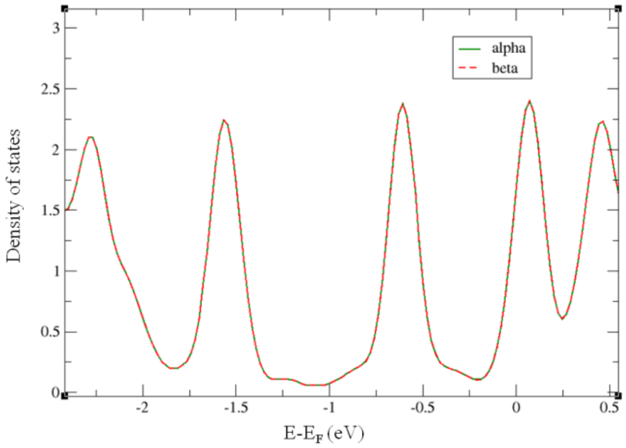

Figure S7. Calculated density of states of **2** on Au(111). The peak positions are -2.27, -1.57, -0.61, 0.07 and 0.47 eV, to be compared with the molecular orbital energies in gas phase (referred to the HOMO energy) of -1.81, -0.67, 0 and 0.3 eV.

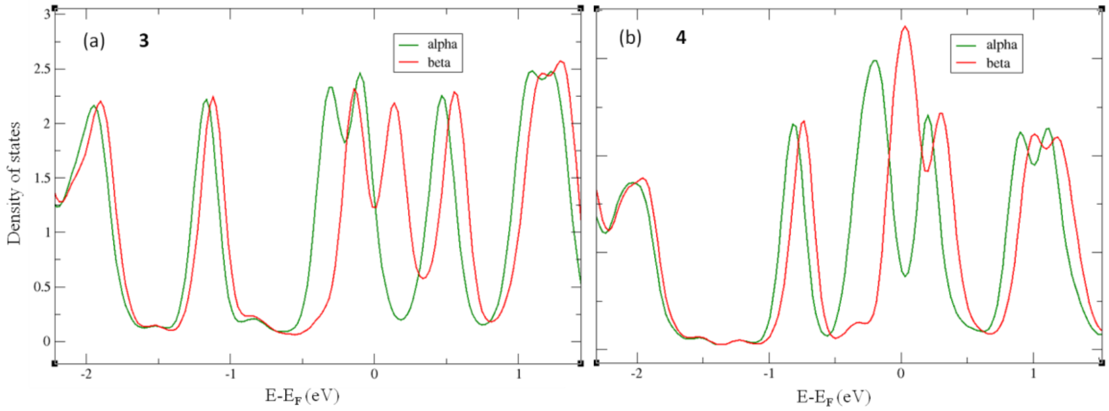

Figure S8. Calculated density of states of (a) **3** and (b) **4** on Au(111). For molecule **3**, the peak positions for α and β electrons are the following. α: -1.95, -1.17, -0.31, -0.1, 0.47 eV; β: -1.90, -1.12, -0.14, 0.14, 0.56 eV. To be compared with the molecular orbital energies in gas phase (referred to the HOMO energy) of -1.35, -0.34, 0 and 0.35 eV. For molecule **4**, the peak positions for α and β electrons are the following. α: -2.03, -0.82, -0.2, 0.2 eV; β: -1.95, -0.73, -0.03, 0.30, eV. To be compared with the molecular orbital energies in gas phase (referred to the HOMO energy) of -0.84, -0.11, 0 and 0.21 eV.

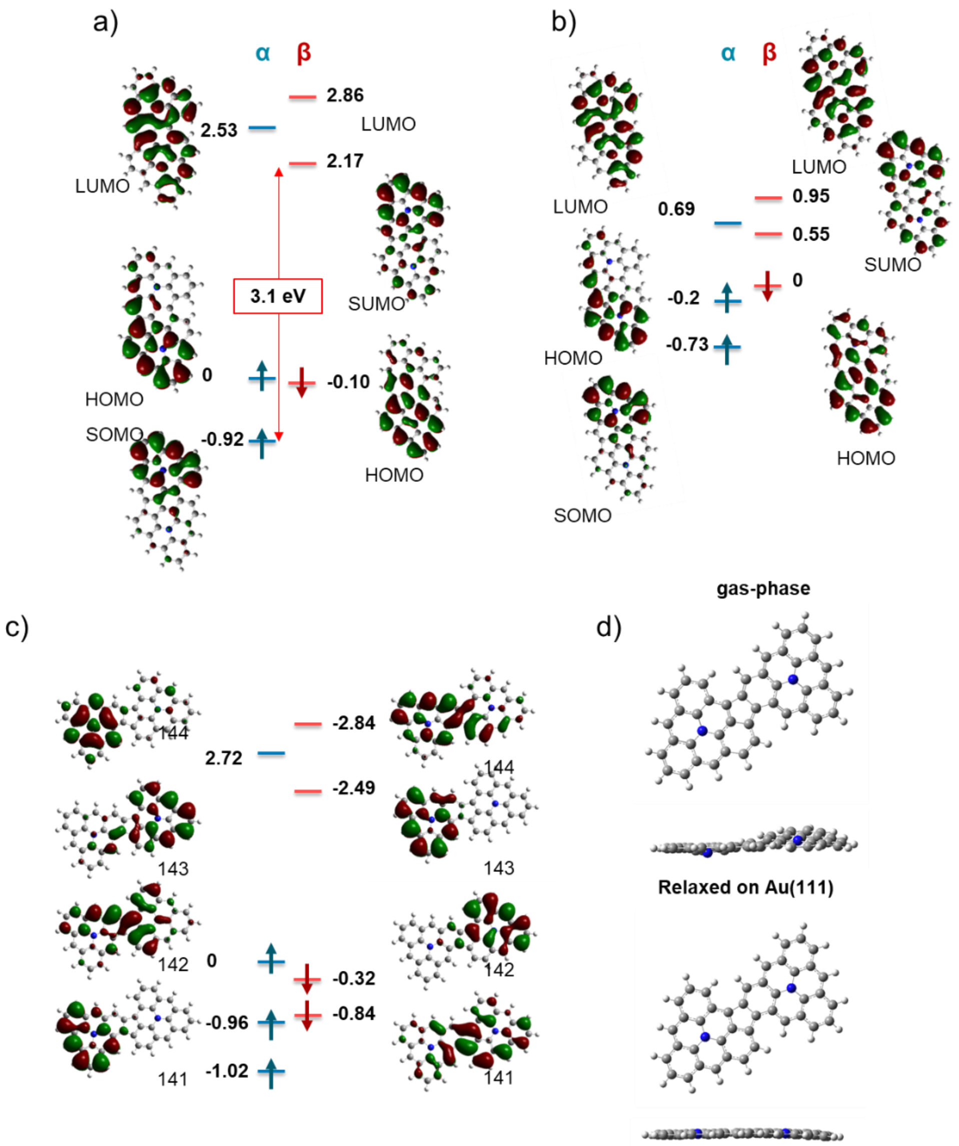

Figure S9. Unrestricted orbitals generated from the optimized structure of **3**$^+$ in (a) gas phase (DFT M062X/6-311g(d,p)) and (b) post-relaxation on a Au(111) slab (See computational details). Energies in eV were normalized to the HOMO. The shapes of the orbitals are mostly similar except for the HOMOβ, probably due to small changes in structure upon relaxation on the surface. Note that the orbitals in (b) were used for the calculation of the simulated dI/dV maps. (c) Gas-phase orbitals of **4**$^+$. (d) Top and lateral views on the geometry of 4 in the gas-phase and after relaxation on Au(111)

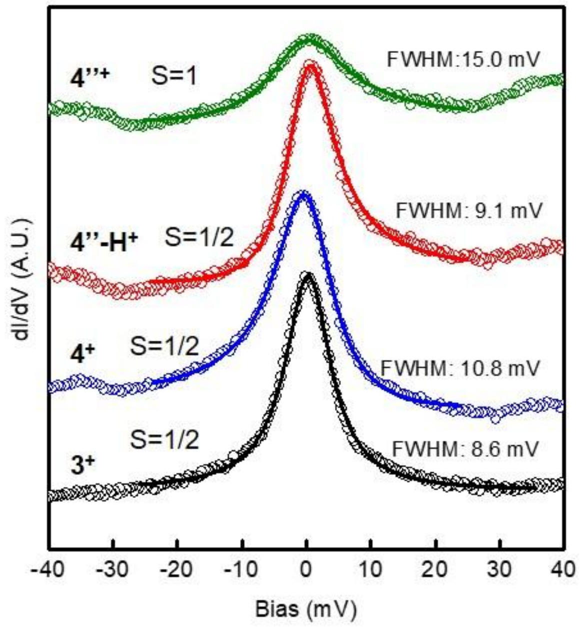

Figure S10. Frota function fitting for Kondo peaks obtained on molecules **3**$^{+}$, **4**$^{+}$, **4''**-H^{+}, and **4''**$^{+}$. Full width at half maximum (FWHM) and total spin are marked for each case.

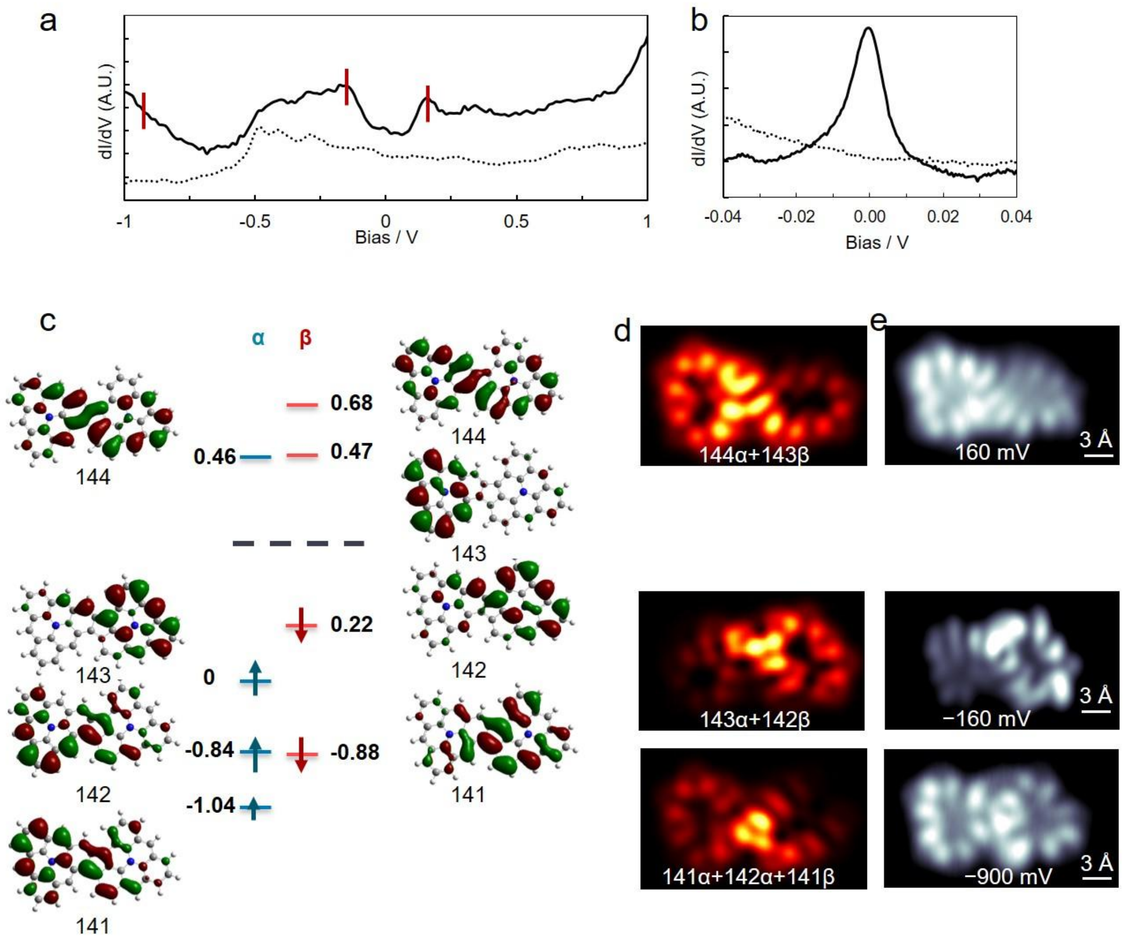

Figure S11. (a) Long-range and (b) low-energy dI/dV spectra of $\mathbf{4}^+$ (solid lines) on Au(111) (dotted line). (c) Gas-phase molecular orbitals of $\mathbf{4}^+$ generated from a single-point calculation on the surface-optimized structure of the molecule (see computational details). (d) Simulated dI/dV maps of the given orbitals, by modelling a CO-functionalized tip. (e) dI/dV maps of $\mathbf{4}^+$ at the given energies obtained in experiment.

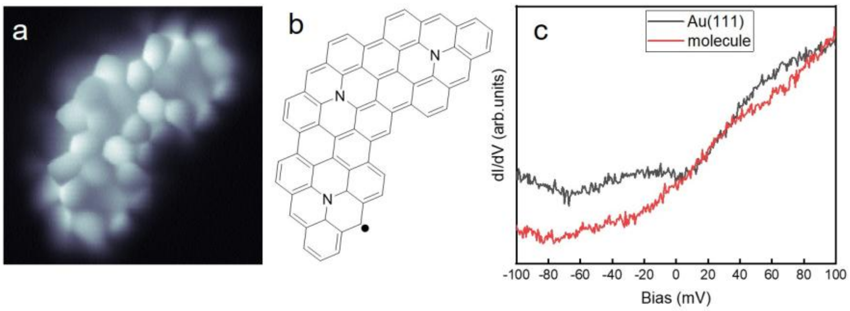

Figure S12. During the course of the experiments, a small number of trimeric structures were found. A "symmetric" structure, in the sense that the reactive zigzag edges of a monomer are connected to reactive zigzag edges of another, is shown here. (a) Bond-resolving STM image of the fused symmetric aza-triangulene trimer (5 mV; constant height mode), whose chemical structure is shown in (b). This structure has an odd number of π-electrons, as explicitly shown. However, the (c) dI/dV spectra obtained on the Au(111) surface showed no magnetic fingerprints (e.g. Kondo resonance, spin flip excitations). Similarly as the monomer and dimers, this can be explained by the transfer of an electron from the molecule to the Au(111) substrate.

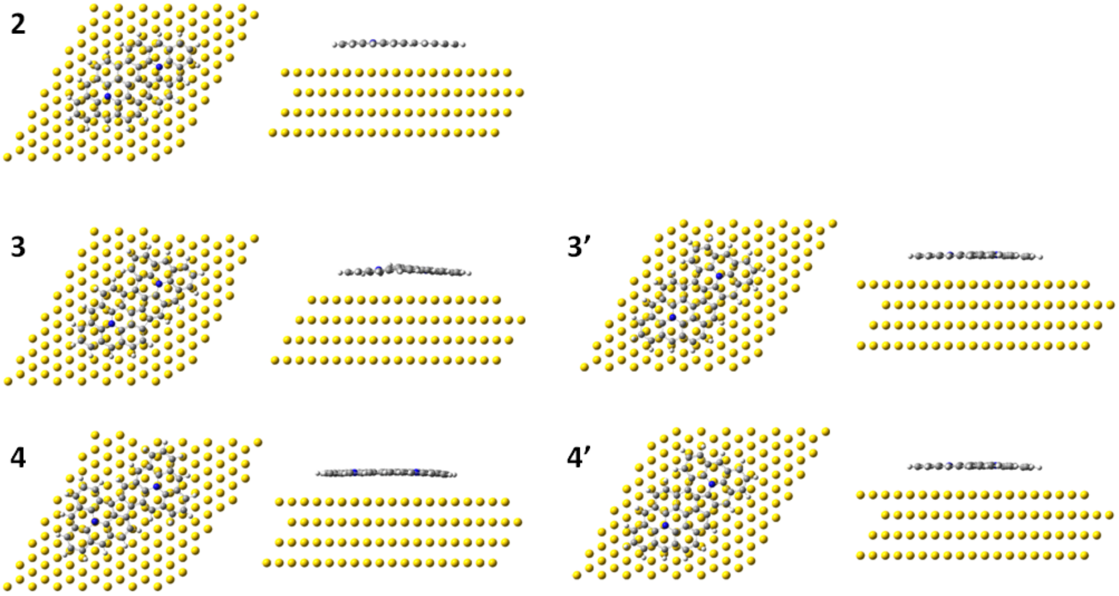

Figure S13. Relaxed 3D structure for molecules adsorbed on Au(111).

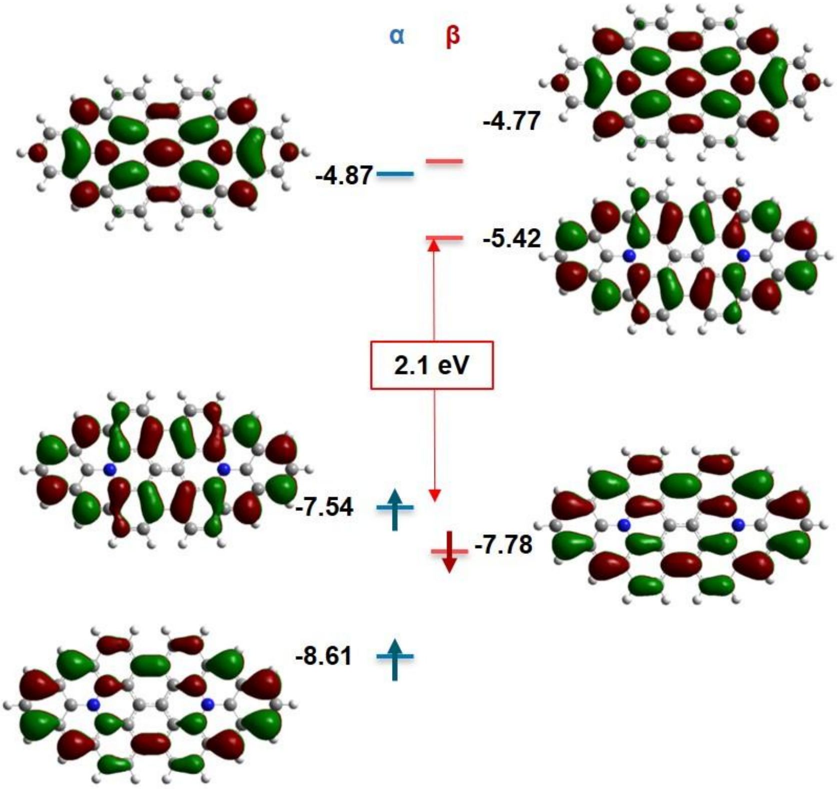

Figure S14. Unrestricted gas-phase orbitals for $\mathbf{2}^{+}$.

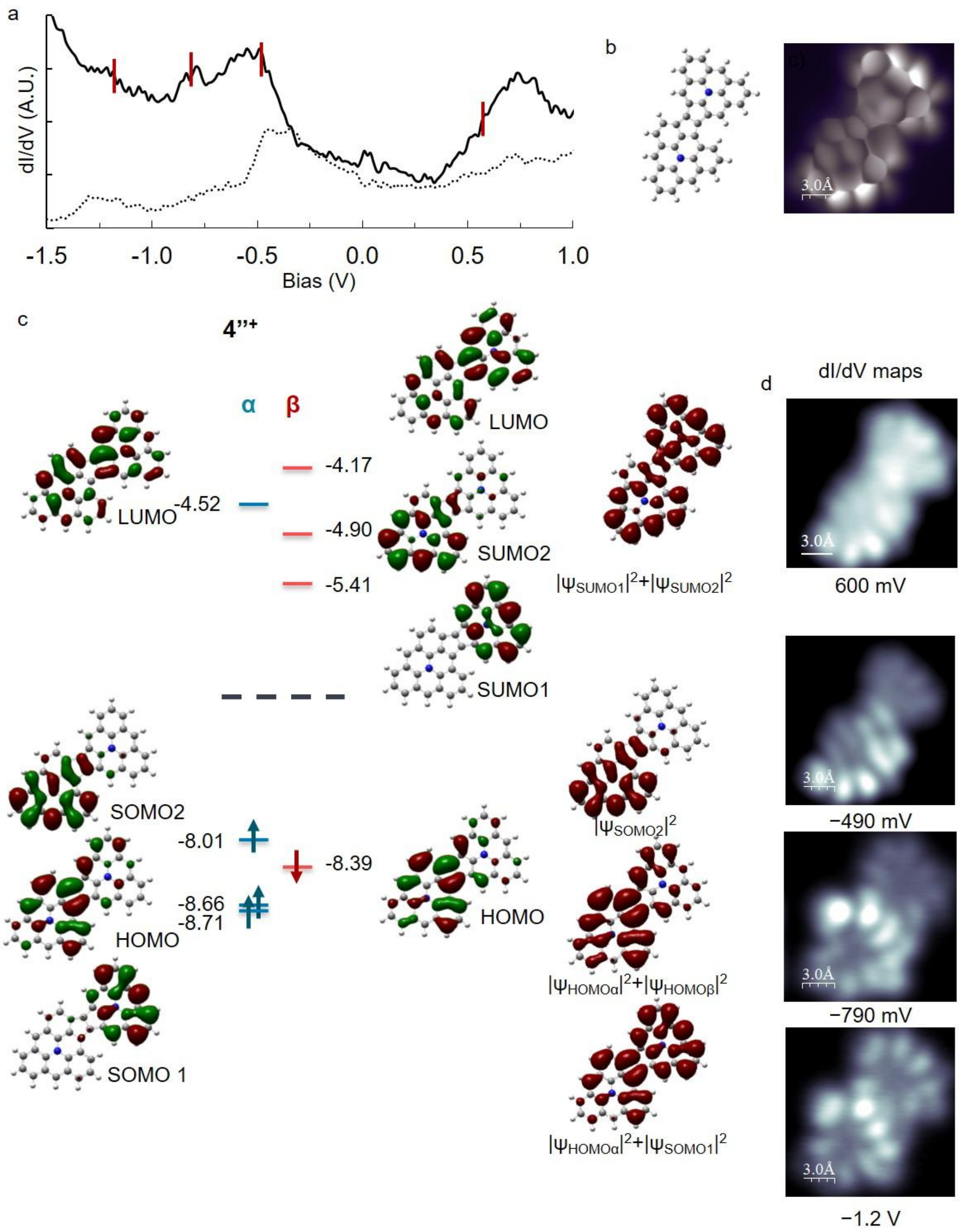

Figure S15. (a) Long-range STS of **4''**$^+$ on Au(111). (b) Molecular orbitals and (c) DOS in the gas phase of **4''**$^+$. (d) dI/dV maps of **4''**$^+$ at the given energies.

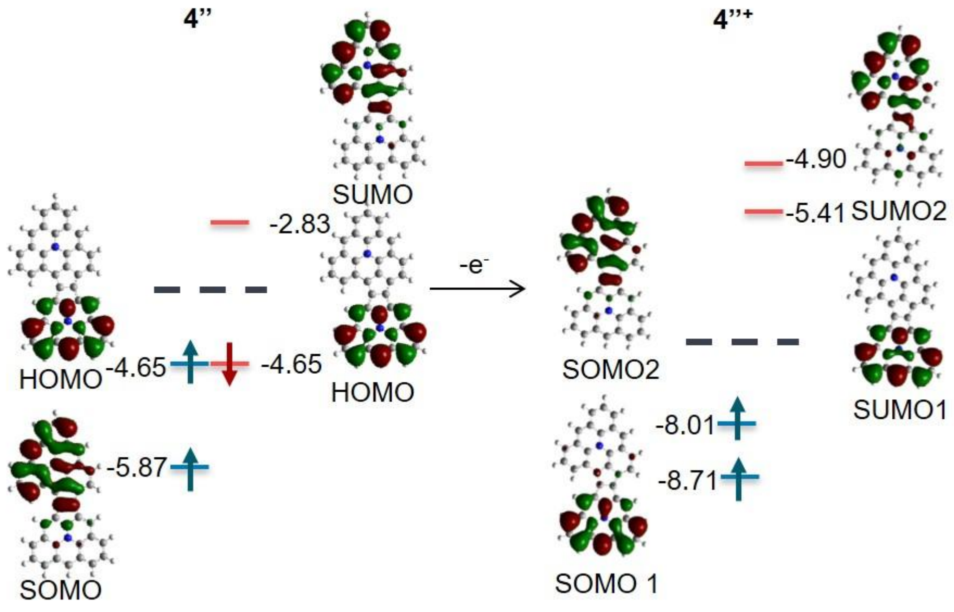

Figure S16. Frontier molecular orbitals of **4''** before and after charge transfer (M06-2x/6-311g(d,p)).

Supplementary Table

Table S1. Details of number of molecules observed upon heating Au(111) to 300 °C.

	Number
**1**	67
**1** (incompletely reduce)	75
Symmetric dimers (**2**)	55
Rhombic dimers (**3**, **3'**)	22
Trapezoidal dimers (**4**, **4'**, **4''**)	15
Total dimers	92
Other trimers[b]	18
Total number of monomer units[c]	380
Theoretical number of dimer[d]	190
Dimer yield	48 %

Methods

STM measurements

STM measurements were performed using a commercial Scienta-Omicron LT-STM at 4.3 K. The system consists of a preparation chamber with a typical pressure in the low 10^{-10} mbar regime and a STM chamber with a pressure in the 10^{-11} mbar range. The Au(111) and Ag(111) crystal was cleaned *via* two cycles of Ar^+ sputtering and annealing at 720 K. The molecular dimers were synthesized on surfaces following the protocol for the synthesis of aza-triangulene monomer[1] with a further annealing at higher temperatures (570 K on Au(111) and 620 K on Ag(111)).

To obtain BR-STM images, the tip was functionalized with a CO molecule that was picked up from the metal surfaces. CO was dosed into the STM chamber *via* a leak valve at a pressure of approximately 1×10^{-8} mbar. For CO adsorption onto the sample, the STM thermal shields were opened and closed again when reaching a sample temperature of 7.0 K. Three such shield opening cycles were typically applied. CO can be picked up with a metallic tip by scanning with a high current and negative bias (*e.g.* I = 1 nA, U = −0.5 V). dI/dV measurements were recorded with the internal lock-in of the Nanonis electronics. The oscillation frequency used in experiments is 797 Hz and the amplitude is 20 mV for long-range dI/dV spectra and maps and 2 mV for low-energy dI/dV spectra shown in the manuscript.

DFT calculations

Gas phase DFT calculations were performed using the Gaussian 16 package.[2] Results were visualized by using the software Gaussview[3] and the densities of states were generated using the *cubman* module. The M062x functional and 6-311g(d,p) basis set were initially used as in previous studies of aza-triangulene[1,4] and other heteroatom-doped triangular graphene flakes.[4] The closed-shell nature of neutral species were confirmed by checking the stability of the wavefunction using the *stable=opt* keyword.

Spin-polarized periodic DFT calculations were performed with the Vienna Ab initio Simulation Package (VASP) software,[5,6] using the OPTPBE functional.[7] The electron density was expanded in a plane-wave basis set up to a kinetic cutoff of 400 eV. The interaction between electrons and nuclei was described with the Projected Augmented Wave (PAW) pseudopotentials, as provided by the VASP database. Reciprocal space was sampled using the Γ-point.

To model the Au(111) surface, a 4-layer slab was used, which contains 7x7 atoms per layer (196 Au atoms) and a vacuum of ~12 Å in order to avoid (spurious) interactions with the closest replica.

We imposed a convergence criterion of 10^{-5} eV for the electron density. Structured were considered as converged when all the Hellman–Feyman forces are smaller than 0.01 eV/Å. For these optimizations, we allow to relax the xyz coordinates of all the atoms in the molecule and z of the outermost metal layer.

Topological analysis of the electron density to obtain atomic charges and spin densities were performed with the code developed by Henkelman et al. [8]

Simulated dI/dV maps were obtained with a home-made software, following a methodology similar to the one developed by Jelinek et al.[9,10]

References

(1) Wang, T.; Berdonces-Layunta, A.; Friedrich, N.; Vilas-Varela, M.; Calupitan, J. P.; Pascual, J. I.; Peña, D.; Casanova, D.; Corso, M.; de Oteyza, D. G. Aza-Triangulene: On-Surface Synthesis and Electronic and Magnetic Properties. *J. Am. Chem. Soc.* **2022**, *144* (10), 4522–4529. https://doi.org/10.1021/jacs.1c12618.

(2) *Gaussian 16, Revision C.01, Frisch, M. J.; Trucks, G. W.; Schlegel, H. B.; Scuseria, G. E.; Robb, M. A.; Cheeseman, J. R.; Scalmani, G.; Barone, V.; Petersson, G. A.; Nakatsuji, H.; Li, X.; Caricato, M.; Marenich, A. V.; Bloino, J.; Janesko, B. G.; Gomperts, R.; Mennucci, B.; Hratchian, H. P.; Ortiz, J. V.; Izmaylov, A. F.; Sonnenberg, J. L.; Williams-Young, D.; Ding, F.; Lipparini, F.; Egidi, F.; Goings, J.; Peng, B.; Petrone, A.; Henderson, T.; Ranasinghe, D.; Zakrzewski, V. G.; Gao, J.; Rega, N.; Zheng, G.; Liang, W.; Hada, M.; Ehara, M.; Toyota, K.; Fukuda, R.; Hasegawa, J.; Ishida, M.; Nakajima, T.; Honda, Y.; Kitao, O.; Nakai, H.; Vreven, T.; Throssell, K.; Montgomery, J. A., Jr.; Peralta, J. E.; Ogliaro, F.; Bearpark, M. J.; Heyd, J. J.; Brothers, E. N.; Kudin, K. N.; Staroverov, V. N.; Keith, T. A.; Kobayashi, R.; Normand, J.; Raghavachari, K.; Rendell, A. P.; Burant, J. C.; Iyengar, S. S.; Tomasi, J.; Cossi, M.; Millam, J. M.; Klene, M.; Adamo, C.; Cammi, R.; Ochterski, J. W.; Martin, R. L.; Morokuma, K.; Farkas, O.; Foresman, J. B.; Fox, D. J. Gaussian, Inc., Wallingford CT, 2016.*

(3) *GaussView, Version 6, Dennington, Roy; Keith, Todd A.; Millam, John M. Semichem Inc., Shawnee Mission, KS, 2016.*

(4) Sandoval-Salinas, M. E.; Carreras, A.; Casanova, D. Triangular Graphene Nanofragments: Open-Shell Character and Doping. *Phys. Chem. Chem. Phys.* **2019**, *21* (18), 9069–9076. https://doi.org/10.1039/C9CP00641A.

(5) Kresse, G.; Furthmüller, J. Efficient Iterative Schemes for *Ab Initio* Total-Energy Calculations Using a Plane-Wave Basis Set. *Phys. Rev. B* **1996**, *54* (16), 11169–11186. https://doi.org/10.1103/PhysRevB.54.11169.

(6) Kresse, G.; Furthmüller, J. Efficiency of Ab-Initio Total Energy Calculations for Metals and Semiconductors Using a Plane-Wave Basis Set. *Comput. Mater. Sci.* **1996**, *6* (1), 15–50. https://doi.org/10.1016/0927-0256(96)00008-0.

(7) Román-Pérez, G.; Soler, J. M. Efficient Implementation of a van Der Waals Density Functional: Application to Double-Wall Carbon Nanotubes. *Phys. Rev. Lett.* **2009**, *103* (9), 096102. https://doi.org/10.1103/PhysRevLett.103.096102.

(8) Tang, W.; Sanville, E.; Henkelman, G. A Grid-Based Bader Analysis Algorithm without Lattice Bias. *J. Phys. Condens. Matter* **2009**, *21* (8), 084204. https://doi.org/10.1088/0953-8984/21/8/084204.

(9) Hapala, P.; Kichin, G.; Wagner, C.; Tautz, F. S.; Temirov, R.; Jelínek, P. Mechanism of High-Resolution STM/AFM Imaging with Functionalized Tips. *Phys. Rev. B* **2014**, *90* (8), 085421. https://doi.org/10.1103/PhysRevB.90.085421.

(10) Hapala, P.; Temirov, R.; Tautz, F. S.; Jelínek, P. Origin of High-Resolution IETS-STM Images of Organic Molecules with Functionalized Tips. *Phys. Rev. Lett.* **2014**, *113* (22), 226101. https://doi.org/10.1103/PhysRevLett.113.226101.
